# Operationalisation of the Randomized Embedded Multifactorial Adaptive Platform for COVID-19 trials in a low and lower-middle income critical care learning health system.

**DOI:** 10.12688/wellcomeopenres.16486.1

**Published:** 2021-01-28

**Authors:** Diptesh Aryal, Abi Beane, Arjen M. Dondorp, Cameron Green, Rashan Haniffa, Madiha Hashmi, Devachandran Jayakumar, John C. Marshall, Colin J. McArthur, Srinivas Murthy, Steven A. Webb, Subhash P. Acharya, Pramodya G. P. Ishani, Issrah Jawad, Sushil Khanal, Kanchan Koirala, Subekshya Luitel, Upulee Pabasara, Hem Raj Paneru, Ashok Kumar, Shoaib Siddiq Patel, Nagarajan Ramakrishnan, Nawal Salahuddin, Mohiuddin Shaikh, Timo Tolppa, Ishara Udayanga, Zulfiqar Umrani

**Affiliations:** 1 Critical Care and Anaesthesia, Nepal Mediciti Hospital, Lalitpur, Bagmati Pradesh, 44600, Nepal; 2 Critical Care, Mahidol Oxford Tropical Medicine Research Unit, Bangkok, Central Thailand, 10400, Thailand; 3 Nuffield Department of Clinical Medicine, University of Oxford, Oxford, UK; 4 Australian and New Zealand Intensive Care Research Centre, School of Epidemiology and Preventive Medicine Monash University, Melbourne, Victoria, Australia; 5 Department of Critical Care, Ziauddin University, Karachi, Sindh, Pakistan; 6 Chennai Critical Care Consultants, Chennai, Tamil Nadu, 600 040, India; 7 Critical Care Medicine, Apollo Specialty Hospital OMR, Chennai, Tamil Nadu, India; 8 The Keenan Research Centre for Biomedical Science, St Michael’s Hospital, Toronto, Ontario, Canada; 9 Department of Critical Care Medicine, Auckland City Hospital, Auckland, New Zealand; 10 Medical Research Institute of New Zealand, Wellington, New Zealand; 11 Faculty of Medicine, University of British Columbia School of Medicine, Vancouver, British Columbia, Canada; 12 School of Medicine and Pharmacology, University of Western Australia, Crawley, Western Australia, Australia; 13 St John of God Hospital, Subiaco, Western Australia, Australia; 14 Critical Care Medicine, Tribhuvan University Teaching Hospital, Kathmandu, Bagmati Pradesh, 44600, Nepal; 15 National Intensive Care Surveillance- MORU, Borella, Colombo, Western Province, 08, Sri Lanka; 16 Critical Care Medicine, Grande International Hospital, Kathmandu, Bagmati Pradesh, 44600, Nepal; 17 Nepal Intensive Care Foundation, Kathmandu, Bagmati Pradesh, Nepal; 18 Pulmonary and Critical Care, Hospital for Advanced Medicine and Surgery, Kathmandu, Bagmati Pradesh, Nepal; 19 Department of Chest Medicine and Critical Care, Ziauddin University, Karachi, Sindh, Pakistan; 20 South East Asian Research in Critical care and Health, Remedial Centre Hospital, Karachi, Sindh, Pakistan; 21 Pulmonary & Critical Care Medicine, National Institute of Cardiovascular Diseases, Karachi, Sindh, Pakistan; 22 Office of Research, Innovation & Commercialization (ORIC), Zuiddin University, Karachi, Pakistan

**Keywords:** Pandemic, clinical trials, research network, registry platform, LMICS, capacity building.

## Abstract

The Randomized Embedded Multifactorial Adaptive Platform (REMAP-CAP) adapted for COVID-19) trial is a global adaptive platform trial of hospitalised patients with COVID-19. We describe implementation in three countries under the umbrella of the Wellcome supported Low and Middle Income Country (LMIC) critical  care network: Collaboration for Research, Implementation and Training in Asia (CCA). The collaboration sought to overcome known barriers to multi centre-clinical trials in resource-limited settings. Methods described focused on six aspects of implementation: i, Strengthening an existing community of practice; ii, Remote study site recruitment, training and support; iii, Harmonising the REMAP CAP- COVID trial with existing care processes; iv, Embedding REMAP CAP- COVID case report form into the existing CCA registry platform, v, Context specific adaptation and data management; vi, Alignment with existing pandemic and critical care research in the CCA. Methods described here may enable other LMIC sites to participate as equal partners in international critical care trials of urgent public health importance, both during this pandemic and beyond.

## Introduction

### COVID-19 in LMICs

A truly global pandemic, the COVID-19 pandemic has placed unprecedented strain on national health systems globally, with demand for intensive care beds and mechanical ventilators rapidly outstripping their availability, even in relatively highly resourced settings. The consequences for low- and middle-income settings, where the quality and availability of health care and related resources (including oxygen, and mechanical ventilation) is typically poorer and where limited pre-pandemic data is available to inform priorities for research, are potentially devastating ^1^. With steroids and possibly IL6 receptor antagonists the exceptions, survival from severe COVID-19 remains dependent on providing the best possible supportive care. Recommendations for supportive therapies are derived mainly from resource-rich settings, whereas recommendations for resource-poor settings in low- and middle-income countries (LMICs), or for rich countries with health systems overwhelmed by the pandemic, are largely lacking ^2^.

### Barriers to clinical trials in LMICs

Developing countries represent the majority of the world’s population and host nearly 90% of the worldwide burden of disease, with communicable diseases including lower respiratory tract infections the prevailing causes for loss of life and disability in low socio-demographic Index (SDI) countries ^1^. High quality clinical research, codesigned and conducted in low SDI countries and in resource constrained LMICS is required in order to discover or verify both the efficacy and effectiveness of treatments in diverse settings. Despite this, LMICs remain severely underrepresented in clinical research - and specifically in clinical trials ^3^. Those stakeholders from LMICs seeking to undertake research often face barriers to successful trial participation; limited expertise or infrastructure to conduct research, lack of financial and human resources and lack of peer to peer support, and complex ethical and regulatory processes- which to navigate costs both time and energy for already overburdened clinicians ^4^.

### REMAP: A platform trial

REMAP-CAP, the Randomised Embedded Multifactorial Adaptive Platform Trial in Community-Acquired Pneumonia (ClinicalTrials.gov ID NCT02735707), is a trial that studies multiple interventions simultaneously and sequentially for patients with severe community acquired pneumonia (CAP) admitted to the intensive care unit (ICU). Its adaptive statistical methods including response-adaptive randomisation enable rapid uptake of study results, meaning patients may benefit from the accruing data. Following the COVID-19 outbreak, REMAP-CAP activated its pandemic mode to focus on treatments for COVID-19 disease in the most critically ill patients. Since the global pandemic in March 2020 REMAP has increased its sites five-fold, internationally.

### Collaboration for Research, Implementation and Training in Critical Care in Asia (CCA)

Wellcome - Mahidol Oxford Tropical Medicine Research Unit supported CCA is a LMIC-led collaborative spanning nine Asian countries ^5^. Its goal is to improve outcomes for critically ill patients, using near real-time high-quality data captured through a cloud-based registry platform to enable setting prioritised improvement, training and research. At its core, the CCA brings together clinical, research, data science and quality improvement stakeholders, who together work to support fledgling acute and critical care registries ^6, 7^. The CCA connects over 95 intensive care units from institutions to provide diverse high-quality data to generate evidence and feedback in near real time for service improvement and research, akin to the foundations of a learning health system (LHS) ^8^.

### Aim

We leveraged the CCA registry platform and existing infrastructure to operationalise REMAP-CAP for COVID and non COVID domains in LMIC ICUs. We sought to overcome the known barriers to operationalising clinical trials in LMICs. Six key aspects of this process are described: i) strengthening an existing community of practice; ii) enabling remote study site recruitment, training and support; iii) harmonising the REMAP trial with existing care processes; iv) embedding REMAP into the existing CCA registry platform; v) providing context specific adaptation and data management, vi) aligning with existing pandemic and critical care research in the CCA.

## Approach

### Strengthening an existing community of practice

We leveraged existing knowledge and expertise within the CCA and adopted a “learning by doing” approach. Stakeholders who included clinicians, researchers, microbiologists, policy makers and laboratory/ procurement services met online to discuss the REMAP-CAP vision and the feasibility of specific trial domains. The national leads and REMAP-CAP International Trial Steering Committee (ITSC) jointly designated the CCA registry team as the central hub for site recruitment training and data coordination in the network sites, providing local ownership and engagement. A regional management committee (CCA RMC) was proposed, consisting of national Principal Investigators (PIs) and members of the CCA from three CCA countries (India, Nepal and Pakistan) where CCA supported critical care registries are now well established and where governance pathways for data collection and sharing as part of the CCA is already established ^6, 7^.

National PIs then together with the CCA RMC and ITSC reviewed the trial protocols and identified potential country and site specific ethical and regulatory bottlenecks. Network members with expertise in navigating ethical and regulatory approval for trials in Asia, provided templates for ethical applications, along with past reviewer questions and the corresponding responses, enabling system memory, shared learning and reducing the burden/ duplication of work for local investigators. Existing REMAP-CAP core protocol documentation and appendices for each domain provided modular, stepwise resources that were presented to national regulatory committees, providing context and precedent for the REMAP trial. The iterative approach process allowed for learning a great deal about trial implementation in diverse regions while ensuring local ownership and engagement was a major focus.

### Remote site onboarding

The already-established CCA registry team—project coordinators, research assistants and data analysts, in partnership with national PI’s, clinical leads, site level data collectors and clinical research nurses—were essential to operationalize REMAP. Stakeholders in existing CCA sites keen to participate in the trial were approached. Previous trial participation was not a requirement, but an established track record of registry data capture for evaluating existing care was. The CCA team, who already provide support to sites in the network with data quality, curation ^9^, and analysis, worked closely with the international REMAP trial team to train the multidisciplinary site level study teams in patient recruitment, consent and study monitoring protocols. The training was delivered online using audiovisual communication mechanisms and leveraged existing international REMAP-CAP resources of training videos. Following training, the central CCA team monitors ongoing trial recruitment and reporting from each site, providing remote support for troubleshooting for technical or research issues. Weekly meetings with the national coordinators using the logs (as described above) offers an opportunity to feedback site level learning and monitor protocol deviations, which are reported through the registry platform. Leveraging existing CCA remote support and communication channels for trial coordination has been especially important given both current COVID pandemic and the geographic distance that the CCA covers.

### Harmonisation with existing care processes

The REMAP design is that the trial research activities and existing clinical care are synergistic. This design aims to streamline research processes and embed them within existing care. This design approach is especially important both in resource constrained health care settings, and during pandemics where trial coordination and research specific activities can at best place additional burden on already exhausted healthcare providers and at worst distract or divert clinicians away from front line care. Two domains; anticoagulation for COVID-19 patients and Vitamin C for severe respiratory illness patients were selected based on their synergy with population specific research priorities, their existing use in clinical care of critically ill COVID and non-COVID respiratory illness patients, and existing availability of the therapeutic agents. National leads and the CCA RMC then worked with site level stakeholders to ensure trial therapeutics and bioclinical sampling within existing supply chain and laboratory services. Trial domains and data points were reviewed, and all (currently 12) recruited centers selected have the necessary laboratory services in place, removing the need for additional trial-specific laboratory resources. Treatments within the two selected domains are already available in the participating centers, meaning additional drug procurement, shipping and storage for the trial was not needed.

### Embedding REMAP into the existing CCA registry platform

**Figure 1.**
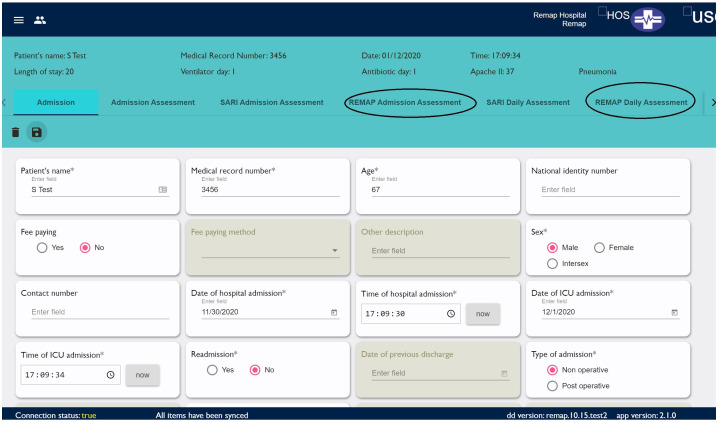
Integration of the REMAP trial domain specific Case Report Forms (CRF) into the CCA registry.

The REMAP-CAP and CCA teams have integrated the REMAP trial domain specific Case Report Forms (CRF) into the CCA registry, ensuring interoperability of data through harmonisation with existing REMAP data structure and CRF logic ( Figure 1). To achieve this, the domain specific CRFs have been mapped to existing case mix, disease severity and prediction, clinical outcomes, and processes of care data points already captured as part of the CCA registry platform.

**Figure 2.**
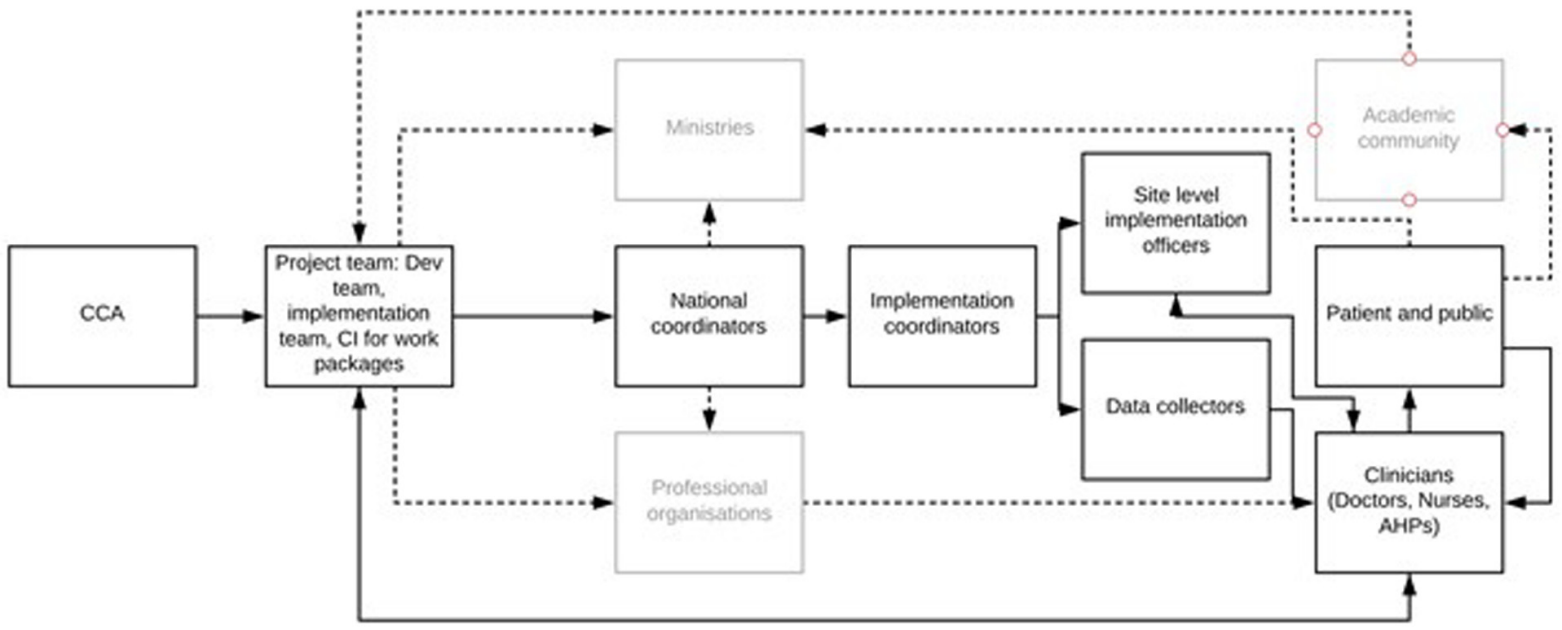
CCA team structure and organogram. Critical care network: Collaboration for Research, Implementation and Training in Asia team (CCA) structure.

To facilitate patient screening, enrollment, and consent, the REMAP CCA has added automated alerts in the participating sites for all severe acute respiratory illness patients as they are entered into the registry alerting data collectors and the research team of a patient's potential eligibility for the trial. The patient eligibility and randomization—arguably one of the most complex aspects of the adaptive trial design—is undertaken in the existing REMAP spinnaker system. The randomisation platform ^10^, embedded in the CCA registry platform, provides a seamless transition for the bedside research team. Consent forms, study protocols, and participatory information leaflets including contact information for the local, national, and international study teams are all available to view, download and print by registered users from the CCA registry platform. Consent forms, once signed are then photographed and uploaded to the CCA platform, placing all study resources and documentation in a central location, where issues pertaining to enrollment and consent can also be reported to and monitored by the central REMAP CCA team, ensuring Good Clinical Practice criteria are met ( Figure 2).

REMAP enrollment completion generates an alert for the central REMAP CCA team, who are then available remotely to support the site level team throughout the study process from enrollment, consent, to patient encounter completion. Once enrolled in the trial, monitoring is all reported through the CCA platform. Existing form structure in the CCA platform follows an ontological approach which mirrors the patient’s journey and prompts the study team to complete forms sequentially. To further ensure in-trial adherence with protocols, successful enrollment of a patient is visible on the patient’s banner bar on their ‘encounter home page’ that indicates the patient’s status, as a context specific adaptation to improve data management of trial participants. Protocol deviations and adverse events are reported through the web-based forms which are then emailed directly to the international REMAP trial coordination team.

### Context specific adaptation and data management

The existing CCA registry data structure allows for site specific variation, with a common data dictionary to enable transformation of data before export ^11^. Study variables such as laboratory tests are reported in the intensive care units’ routine unit of measurement. Terminology and data collection time points have been harmonised with already proven registry data collection practices. The CCA platform’s existing internal data quality mechanisms- field completeness, value range validity and branching logic- all reported through descriptive analytic dashboards promote data quality by prompting users towards completeness and alerting to missed or potential spurious responses. Similarly logs of screening, patient recruitment and study monitoring are visible to the REMAP CCA team national trial leads and the international REMAP project coordinator. 

**Figure 3.**
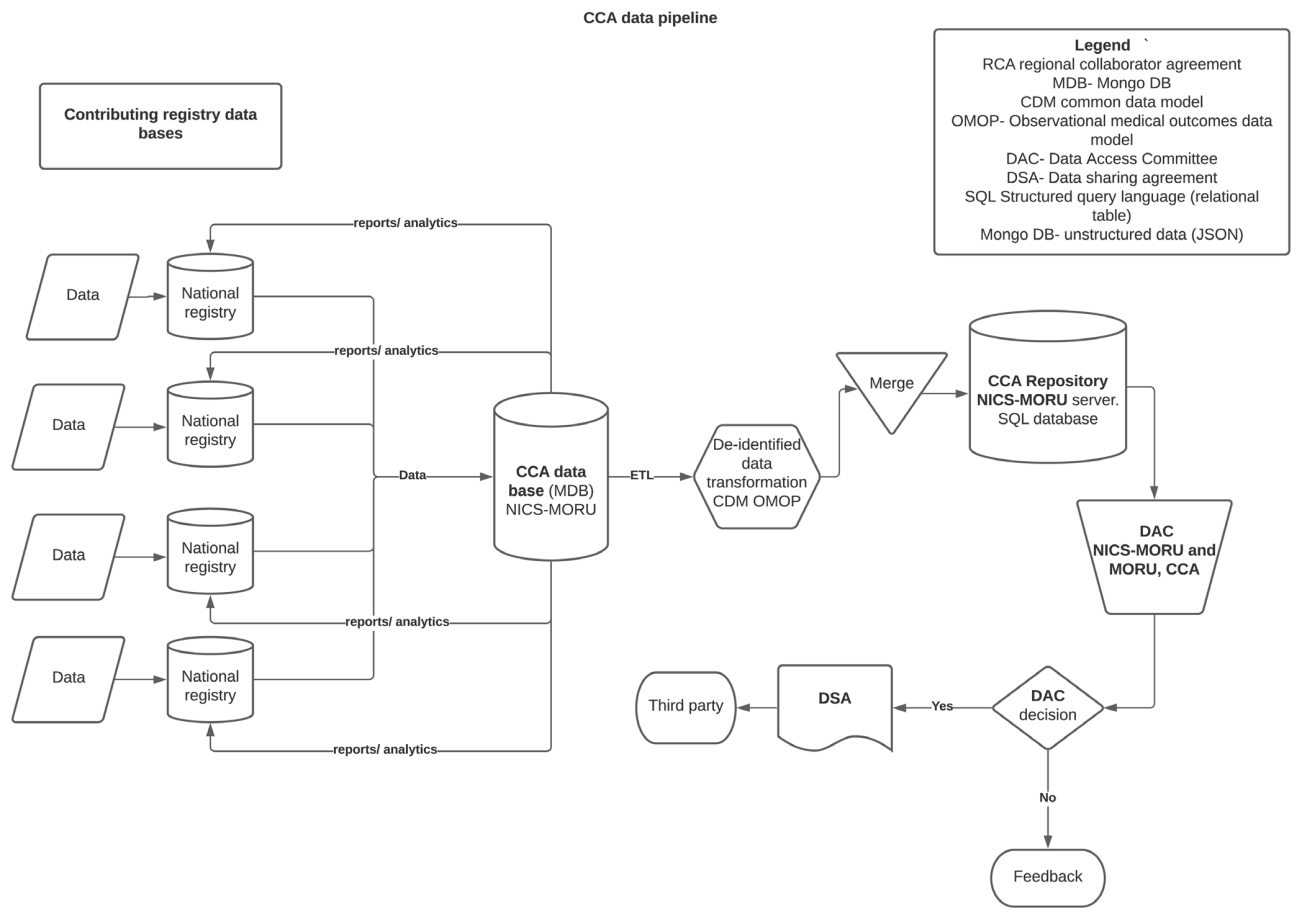
CCA data platform structure.

The registry provides a federated system for data storage, whereby existing national registries house their data- essential for compliance with the national data security requirements ( Figure 3). All anonymised registry and trial data is backed up to a central CCA server. Trial-relevant data are continuously and automatically extracted from the CCA registry platform, and curated into a dataset suitable for export to the international data coordinating center at Monash University. Data are stored in a MongoDB, processed using a scheduled combination of Javascript, and formatted and exported for trial reporting. An unblinded investigator reviews initial exports in detail to ensure data accuracy. Variables that depend on more than one discrete CRF field (e.g. severity of illness score) are reported as raw values, enabling the international statistical analysis committee to validate source data prior to analysis. Data extracts are reviewed by an unblinded investigator and two programmers. Data once extracted is transferred via a secure file transfer service.

### Alignment with existing pandemic and critical care research in the CCA

The COVID-19 pandemic has resulted in unprecedented mobilisation of ministries, policy makers and clinicians to engage in research. In partnership with ISARIC and led by collaborating registry PRICE ^7^, the CCA platform has already been adapted to comply with the ISARIC Clinical Characterisation Protocol as part of the coordinated response to pandemic research. Enabled by the CCA platform's modular structure, common data to ISARIC CCP, REMAP trial and critical care surveillance is entered only once by the bedside registry team ^9^. This reduces the burden of data capture and ensures data pertaining to patient care processes for REMAP is also available to the clinical team for site level service evaluation and improvement. The data is then mapped to a common data model (OMOP) ^12^ as part of the CCA platform’s commitment to the FAIR principles of data management ^13^ and creating the potential for sites to contribute to other international observational pandemic research.

## Impact

The CCA’s and collaborating registries shared expertise, track record in remote working and a prior knowledge of overcoming technical and training barriers to registry adaption was a central tenet of overcoming known barriers to trials described above ^14^. Known barriers to trials in resource constrained health systems; technological challenges including internet bandwidth, navigating institutional firewalls, device availability and human computer interaction were mitigated by the infrastructure already established by the CCA. Similarly, the strong partnership of support for real-time troubleshooting already present between the CCA implementation team and collaborating registries was invaluable during initial recruitment and training. Embedding the REMAP trial domain CRF’s into the existing registry platform has reduced the burden of data capture for frontline clinical staff; essential in resource constrained settings, where failure to optimise and reuse data results in at best poor quality of data and at worst misdirects resources from clinical care. The CCAs upfront investment in a codesigned platform, developed in partnership with frontline clinicians and iterated within the diverse health systems in the CCA, has mitigated against known challenges of data availability and quality. In addition, leveraging the cloud-based platform with inbuilt data audit and feedback mechanisms has enabled hospitals within the geographically wide network to be supported by a centralised research team. Data generated through the CCA will enable evaluation and benchmarking of existing care practices for patients with pneumonia in intensive care and may inform quality improvement projects to implement and share best practices within the community of practice; the hallmarks of a learning health system. Nine sites in three countries are now actively recruiting patients to pandemic related REMAP trial domains; India, Nepal and Pakistan. Other sites in the CCA network are seeking collaboration and will be supported.

### Remaining challenges

Whilst the pandemic has accelerated opportunities for international clinical trial collaboration, particularly amongst clinicians, ethics committees, data sharing committees and research review boards are faced with unprecedented numbers of applications for review. Such committees in many LMICs rely heavily on volunteers, and members may have themselves face rising clinical obligations, which coupled with limited experience of complex trials such as REMAP or of navigating international data sharing- may lead to opportunities to engage in research being rejected or delayed. Collaborations such as CCA, along with grant making bodies, have a duty to further Invest in building capacity for such services and in promoting greater compatibility of governance structures.

## Conclusions

Implementation of the REMAP-CAP for COVID trial in the CCA is feasible and facilitated by multidisciplinary collaboration. This investment establishes important groundwork for future learning health system endeavors. This operationalisation of a randomized controlled platform trial in a registry-embedded manner in three countries of a nascent critical care network can be the stimulus for further democratisation of international trial participation.

## Data availability

No data are associated with this article.

## Author information

The first 11 authors contributed equally and are listed alphabetically.
