# Supplementary material for: Operationalisation of the Randomized Embedded Multifactorial Adaptive Platform for COVID-19 trials in a low and lower-middle income critical care learning health system
Source: Wellcome Open Res. 2021 Jan 28;6:14. [Version 1] doi: 10.12688/wellcomeopenres.16486.1 (PMC7883321; doi:10.12688/wellcomeopenres.16486.1)
Supplement: Peer review history

# Peer review: Reviewer response for version 1

**DOI:** 10.21956/wellcomeopenres.18151.r42371

**Published:** 2021-02-16

**Authors:** Michael Lipnick, Sky Vanderberg

**Affiliations:** 1 Center for Health Equity in Surgery and Anesthesia, Department of Anesthesia and Perioperative Care, University of California San Francisco, San Francisco, CA, USA; 2 University of California San Francisco, San Francisco, CA, USA

This article focuses on an important topic in clinical research - the underrepresentation of LMICs in clinical research/trials. This article describes the implementation of the REMAP-CAP study and CCA collaboration as impressive examples of how to promote equitable and context-relevant research.

 Overall the manuscript is excellent though could benefit from some additional copyediting. Please find additional minor comments for consideration and revision below: 

**Introduction:**

- The terms low SDI and LMIC are both used independently and in combination - consider streamlining terminology.
- May be helpful to make the point that interventions designed/implemented for respiratory failure in well-resourced settings have the potential to cause harm when applied in LMICs (i.e. mechanical ventilation) without other considerations in place.
- Consider expanding on the reference/origin for 'known barriers' to operationalising clinical trials in LMICs or expanding on the process that identified the 'six key aspects' of the process.
- When discussing REMAP-CAP trial design, consider further clarifying how study patients benefit from the response-adaptive randomization in near real time.
- Second sentence in Collaboration for Research, Implementation and Training (CCA) section needs clarification.  

**Approach:**

- Strengthening an existing community of practice section: Consider expanding on the lasting positive effects of this endeavor. Specifically, what was learned/gained during this process that will facilitate future inclusion of LMIC sites in international clinical trials.
- Consider expanding on how the CCA model of remote onboarding/monitoring can be replicated elsewhere.
- Harmonisation with existing care processes section: consider clarifying the message in the first sentence.  
- Context specific adaptation and data management section: Consider additional citations to specify the “already proven registry data collection practices.” 

**Impact:**

- First paragraph: Could you present quantifiable data such as percent completeness, any audit data as available, to underscore the degree of impact.
- Remaining challenges section: Consider clarifying the wording of the first sentence, as we do not know which groups are faced with accelerated opportunities vs unprecedented numbers of applications.
- Please expound upon remaining challenges, as bottlenecks at ethics/regulatory committees are likely not the only remaining challenges for this type of work.

** **


**Conclusions: **Could consider also highlighting the scientific merit to include LMIC sites in international critical care clinical trials.

**Figure/Tables:**

**Figure 2. **

- Please consider including figure legend to specify the significance of dotted lines, red circles in “academic community” box, greyed-out boxes, and lines which have arrows in opposite directions (?bidirectional).

**Figure 3. **

- Consider color coding to further delineate data flow in the direction of national registries to CCA database from reports/analytics flow in the direction of CCA data to national registries.
- Define “ETL” in legend.

Does the article adequately reference differing views and opinions?

Yes

Are all factual statements correct, and are statements and arguments made adequately supported by citations?

Yes

Is the Open Letter written in accessible language?

Yes

Where applicable, are recommendations and next steps explained clearly for others to follow?

Yes

Is the rationale for the Open Letter provided in sufficient detail?

Yes

Reviewer Expertise:

ARDS, research in HIC-LMIC collaborations

We confirm that we have read this submission and believe that we have an appropriate level of expertise to confirm that it is of an acceptable scientific standard.

# Peer review: Reviewer response for version 1

**DOI:** 10.21956/wellcomeopenres.18151.r42370

**Published:** 2021-02-12

**Authors:** Elisabeth Riviello

**Affiliations:** 1 Division of Pulmonary, Critical Care and Sleep Medicine, Beth Israel Deaconess Medical Center, Boston, MA, USA; 2 Harvard Medical School, Boston, MA, USA

**Major comments**

 This is a really important and well-structured manuscript, providing a detailed description of how a regional international ICU registry was used to make international trial participation feasible for three LMICs. The detail provided is incredibly helpful as readers think about applying the lessons learned by this group.

 In particular the following descriptions were helpful: use of the community of practice for IRB navigation; using the CCA structure and REMAP resources to onboard remote sites; choosing interventions that are feasible for the resources at the sites in question; embedding every part of the CRF and consent into the CCA registry; and a very detailed description of the data management processes.

 One barrier I would love to see addressed is compensation for time spent, including both PIs as well as data collectors. Often international trials expect sites to provide leadership (PIs) and data collection, with little or no compensation. This is far less feasible in LMICs, where clinicians are already stretched, as noted in the manuscript. A description of how time is compensated (or not) for the baseline registry, and whether the REMAP-CAP trial added resources to this, would be very helpful. If the answer is that compensation is largely not provided, this is also interesting information – and would suggest that the registry is so useful to clinicians that the time spent entering data is considered part of clinical care time. Any detail that can be provided on this would be a welcome addition. However, the authors are certainly not required to add comments on this, as it may not be a relevant learning point from their experience.

 Overall the manuscript could benefit from copy editing, particularly in shortening some lengthy sentences, and adding and deleting commas to improve clarity.

**Minor comments**

 Title:

- If allowed by Wellcome formatting rules, I would add the acronym (REMAP-CAP) after the full name in the title, as this is a well-known trial group, recognized more easily by its acronym name.

Abstract:

- It is not obvious how CCA is the acronym for the group as spelled out (would add back in the “Critical Care” part, for the “CC” of the acronym).
- A dash is not needed between centre-clinical. Instead, there could be a dash in multi-centre.
- Would say “Methods described focus” rather than “Methods described focused”.
- Would use usual numbering: 1) or i) rather than i,
- No need to capitalize the implementation areas described

Introduction

- I would provide citations for: 1) the statement of scarcity of resources even in highly-resourced areas and 2) the mention of steroids and IL-6 inhibitors as evidence-based treatments for COVID-19.
- Would say “bear …” rather than “host …”
- “High quality clinical research … in low SDI countries and in resource constrained LMICs” … are the authors meaning say that low SDI countries and LMICs are distinct from one another? If not, it would be better to use only one of these as the descriptor of resource-limited settings.
- “successful trial participation” should be followed by a colon rather than a semicolon.
- “Those stakeholders from LMICs seeking to undertake …” is a long sentence, and would benefit from shortening, particularly the last parenthetical about the time and energy required for regulatory processes.
- “REMAP: a platform trial”: I would cite the description noted in the first sentence; even citing the REMAP-CAP website would be sufficient.
- “CCA”: I would start the first sentence of this section with “The” before “Wellcome-Mahidol …”.
- “CCA”: I would add a citation for the sentence describing CCA; the website for CCA would be sufficient.

Figure 3:

- This would be even more useful if REMAP-CAP were mentioned, diagramming at what point in the process the data goes to the REMAP-CAP group.

Does the article adequately reference differing views and opinions?

Yes

Are all factual statements correct, and are statements and arguments made adequately supported by citations?

Partly

Is the Open Letter written in accessible language?

Partly

Where applicable, are recommendations and next steps explained clearly for others to follow?

Yes

Is the rationale for the Open Letter provided in sufficient detail?

Yes

Reviewer Expertise:

global health, resource-poor settings, critical care, ARDS, sepsis

I confirm that I have read this submission and believe that I have an appropriate level of expertise to confirm that it is of an acceptable scientific standard.

# Peer review: Reviewer response for version 1

**DOI:** 10.21956/wellcomeopenres.18151.r42367

**Published:** 2021-02-09

**Authors:** Paul Young

**Affiliations:** 1 Medical Research Institute of New Zealand, Wellington, New Zealand

This is a very clear account of an important and impressive piece of work implementing a complex global trial within an existing registry.  It provides a blueprint for future efforts to involve LMICs in international critical care trials. The article is scientifically sound and I have no suggestions for changes except that "data is" should be changed to "data are" because the singular form of data is datum.

Does the article adequately reference differing views and opinions?

Yes

Are all factual statements correct, and are statements and arguments made adequately supported by citations?

Yes

Is the Open Letter written in accessible language?

Yes

Where applicable, are recommendations and next steps explained clearly for others to follow?

Yes

Is the rationale for the Open Letter provided in sufficient detail?

Yes

Reviewer Expertise:

intensive care medicine

I confirm that I have read this submission and believe that I have an appropriate level of expertise to confirm that it is of an acceptable scientific standard.
